# Application of Distributed Optical Fiber Sensing Technology to the Detection and Monitoring of Internal Swelling Pathologies in Massive Concrete Blocks

**DOI:** 10.3390/s22207797

**Published:** 2022-10-14

**Authors:** Ismail Alj, Marc Quiertant, Aghiad Khadour, Quentin Grando, Karim Benzarti

**Affiliations:** 1 Matériaux et Structures (MAST) Department, Expérimentation et Modélisation pour le Génie Civil et Urbain (EMGCU), Univ Gustave Eiffel, F-77447 Marne-la-Vallée, France; 2 Composants et Systèmes (COSYS) Department, Laboratoire Instrumentation, Simulation et Informatique Scientifique (LISIS), Univ Gustave Eiffel, F-77447 Marne-la-Vallée, France; 3 Institut de Radioprotection et de Sûreté Nucléaire (IRSN), CEDEX, 13115 Saint-Paul-Lez-Durance, France; 4 Lab Navier, Univ Gustave Eiffel, Ecole Nationale des Ponts et Chaussées (ENPC), Centre National de la Recherche Scientifique (CNRS), F-77447 Marne la Vallée, France

**Keywords:** Distributed Optical Fiber Sensors (DOFS), Structural Health Monitoring (SHM), reinforced concrete structures, strain measurements, concrete pathologies

## Abstract

This paper presents an experimental application of Distributed Optical Fiber Sensors (DOFS) for the Structural Health Monitoring (SHM) of concrete structures affected by internal swelling pathologies. In the framework of a large research project aiming to assess the possible extension of the operating lifetime of nuclear power plants from 40 to 60 years, massive blocks were cast from reactive concrete mixtures intended to develop delayed ettringite formation and alkali–silica reaction. These blocks were subjected to specific ageing conditions to initiate and accelerate the concrete pathologies. Some of the blocks were instrumented with DOFS bonded to the surface and embedded in the concrete. Using an interrogator device based on Rayleigh backscattering and a suitable procedure to eliminate temperature effects, distributed strain measurements were then performed at different time intervals. The first results of this ongoing study made it possible to demonstrate the feasibility and effectiveness of this sensing technology for detecting and monitoring expansion induced by swelling pathologies in representative-scale concrete structures.

## 1. Introduction

In France, the existing nuclear power facilities were initially designed for a service lifetime of 40 years, and many of them will reach this milestone in the next few years. The main operator and the nuclear safety authorities are considering an extension of this lifetime up to 60 years on a case-by-case basis, invoking both technological advances in the maintenance processes and economic reasons [1,2].

Several important structures of nuclear power plants (such as reactor components, cooling towers, safety, and containment systems, for instance) consist of massive reinforced concrete elements. Over long operating periods, these structures may be affected by concrete disorders resulting from complex chemical processes, such as internal swelling reactions. As a consequence, the mechanical properties and the ability of concrete to ensure the containment of radioactive substances may be reduced. Deep knowledge of the long-term behavior of such critical infrastructures is thus necessary before reconsidering their lifespan, especially when concrete compositions prone to internal swelling pathologies were used during the building phase [3].

In this context, the French Institute for Radioprotection and Nuclear Safety (IRSN) launched the ODOBA project in 2016 (Observatory of the Durability of Reinforced Concrete Structures) [4], with the objective of investigating the internal swelling pathologies of concrete, namely Delayed Ettringite Formation (DEF) and Alkali–Silica Reaction (ASR) [5,6], as well as their consequences on containment buildings of nuclear power plants. The ODOBA program involves the manufacture of representative specimens (massive blocks of plain or reinforced concrete) based on the concrete formulations that were actually used in the construction of existing nuclear power plants. DEF and ASR (considered separately or in combination) are then initiated and accelerated in these concrete blocks. Their effects are monitored over several years using well-established or more exploratory measurement techniques (conventional vibration wire gauges, ultrasonic or other non-destructive methods, etc.), with a view to a better understanding of the key factors governing the development of such pathologies in large-scale specimens. It is also expected to develop and validate numerical tools for predicting the long-term behavior of concrete structures affected by swelling pathologies. The outcomes of this ongoing ODOBA project will contribute to the decision-making process regarding the lifetime extension of nuclear infrastructures.

The work presented in this article is part of the ODOBA project and focuses on metrological aspects, with an emphasis on the deployment of Distributed Optical Fiber Sensors (DOFS) since our team has gained knowledge in this field from previous studies [7,8,9,10,11,12,13]. The main objective was to evaluate the applicability of this technology for monitoring the development of internal swelling pathologies in the massive ODOBA concrete blocks. It is worth noting that semi-distributed optical fiber strain sensor [14,15,16] and DOFS have already proven their effectiveness for the health monitoring of large concrete structures under service conditions (such as bridges, tunnels, dikes, etc.) [17,18,19]. DOFS enable continuous strain/temperature measurements over long distances and with a sub-millimeter spatial resolution. However, to the best of our knowledge, there is no documented attempt to detect or monitor the concrete expansion induced by swelling reactions using such distributed sensors. Scarce applications of long gauge fiber optic extensometers embedded in concrete structures affected by ASR are reported in the literature [20,21], but these sensors provide only a single measurement averaged over the gauge length and can hardly provide information about the health state of the whole structure.

The ODOBA specimens were instrumented with DOFS cables according to procedures developed in previous studies [9,22,23]: some DOFS cables were embedded in the concrete medium during casting, while others were bonded to the external surface of the blocks after fabrication. Distributed strain measurements were then carried out along the sensing line using an interrogator device based on Rayleigh backscattering, both in the reference state and after exposure of the concrete blocks to specific ageing conditions (intended to initiate/accelerate the swelling reactions). A simple procedure was also proposed to remove temperature effects from the strain profiles, and the reliability of the DOFS data was further checked through comparison with measurements from conventional sensors (vibrating wire gauges).

The preliminary results of this experimental campaign are analyzed in this paper to evaluate if the DOFS instrumentation makes it possible (i) to detect and monitor swelling phenomena related to DEF or ASR in large-scale structures and (ii) to conclude on the reactivity of the concrete mix compositions of the various blocks. The first part of the paper is dedicated to the description of the ODOBA concrete blocks and the ageing protocols intended to initiate/accelerate the swelling pathologies. These protocols include exposing the blocks to weather conditions (natural ageing) or controlled environments (accelerated ageing in thermo-regulated water pools). The second part presents the instrumentation scheme of the concrete blocks and the procedures for installing the embedded/bonded DOFS cables. In the last part, the DOFS strain profiles collected on two peculiar blocks subjected to accelerated ageing conditions serve as a basis for the discussion.

## 2. Description of the Concrete Blocks of the ODOBA Project and Their Ageing Conditions

### 2.1. Presentation of the Blocks and Their Conventional Instrumentation (Vibrating Wires and Thermocouples)

**Figure 1:**
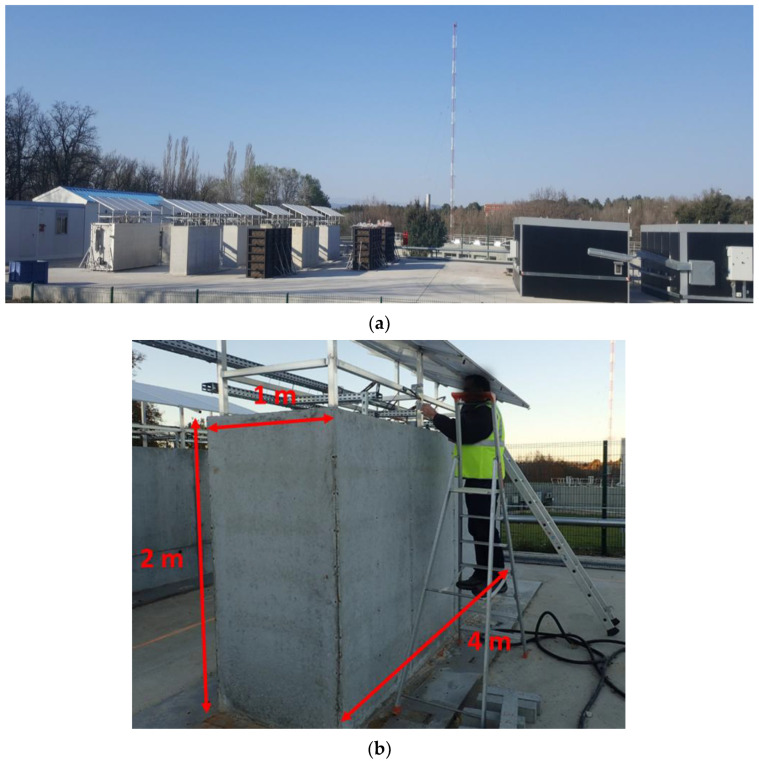
(**a**) Exposure platform for ODOBA blocks, and (**b**) dimensions of the blocks.

The concrete blocks of the ODOBA project are stored on a dedicated outdoor platform in Cadarache (south of France) and arranged in rows (Figure 1a). Each block measures 1 × 2 × 4 m^3^ (Figure 1b) and weighs about 20 tons. These massive blocks are divided into three categories: Reference blocks which are not expected to develop pathologies, potentially pathological blocks subjected to external weather conditions (referred to as “natural ageing” conditions), and potentially pathological blocks subjected to experimental protocols aimed at accelerating the development of pathologies (referred to as “accelerated ageing” conditions).

**Figure 2:**
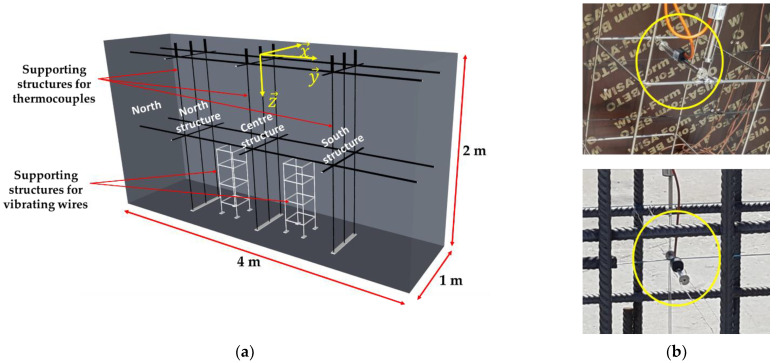
(**a**) Supporting structures for conventional sensors in NRC concrete block; (**b**) Installation of vibrating wires in an NRC block (top) and in an RC block (bottom).

The studied blocks are either reinforced concrete (RC) blocks or non-reinforced concrete (NRC) blocks. To continuously monitor temperature and strain at several locations inside the blocks, thermocouples and vibrating wires (VWs) are embedded in the concrete during the fabrication of the blocks. For NRC blocks, thin metallic supports are used to hold the different sensors (see Figure 2a). Differently, for RC blocks, the sensors are attached directly to the steel reinforcement of the blocks (Figure 2b).

All of the blocks are equipped with a total of six VWs of gauge length 15 cm, model MicroVib from Cementys Company (Palaiseau, France).

Regarding NRC blocks, metallic frames supporting the VWs are installed at two specific locations (Figure 2a). Three VWs are attached to each frame (one VW along each direction$\;\vec{x}$, $\vec{y}$, and $\vec{z}$—see reference coordinate system in Figure 2a), allowing for strain measurements along the three directions of space at each support location.

For the RC blocks (without supporting structures), the VWs are arranged according to the same geometrical configuration but using the steel reinforcement of the block as a supporting frame (Figure 2b bottom).

All of the concrete blocks are also equipped with 53 T-type thermocouples (allowing temperature measurements in the range of −185 °C/300 °C).

**Figure 3:**
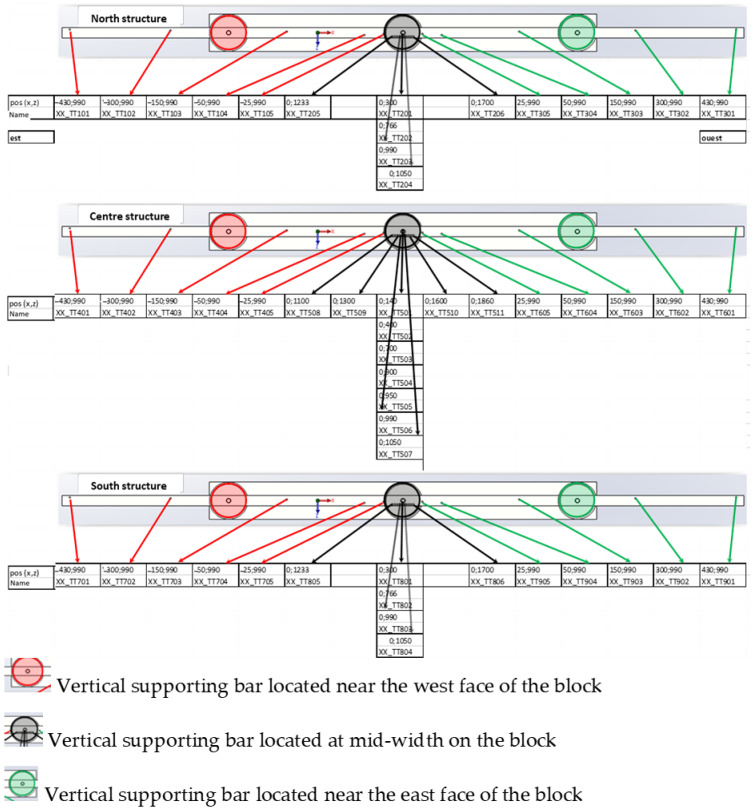
Coordinates (in mm) of the thermocouples in an NRC concrete block considering the origin of the coordinate system at the center of the upper face of the block.

In the case of NRC blocks, these thermocouples are attached to three vertical structures, which are represented in black in Figure 2a. The thermocouples are thus arranged on three vertical ($\vec{x}$, $\vec{z}$) planes: one plane close to the north side of the block, another at the mid-width of the block, and the last plane near the south side of the block. This arrangement provides a global overview of the temperature gradients within the block. The locations of the various thermocouples on the three vertical structures are detailed in the scheme of Figure 3. The coordinates shown in Figure 3 are relative to the reference coordinate system drawn in Figure 2a (origin at the center of the upper face of the block).

In the RC blocks, the thermocouples were installed at the same locations, but the steel stirrups served as a supporting frame.

All of the vibrating wires and thermocouples were connected to a centralized acquisition unit, which collected temperature and strain data every 15 min.

### 2.2. Concrete Pouring, Conditioning of the Blocks and Ageing Protocols

The concrete mix composition of each block and the conditioning procedure were chosen according to the pathology to be studied. Reactive aggregates were added to the concrete mix composition in the case of blocks intended to develop ASR [24]. On the other hand, for blocks intended to develop DEF, the pathology was activated by applying a thermal treatment immediately after concrete pouring, using a specific heating chamber [24,25].

Prior to the pouring of the NRC blocks, the metallic structures intended to support the sensors (Figure 2a) were installed inside the formwork. The sensors were attached to these structures, and the last side of the formwork was then put in place. The whole formwork was finally tightened and hence ready to support the concrete weight.

For the RC blocks, the steel stirrup was assembled first. Then, the different sensors were installed at the defined locations, supported by the steel rebars. Once the sensor setup was finished, the stirrup was inserted inside the formwork. Finally, concrete was poured into the formwork.

**Figure 4:**
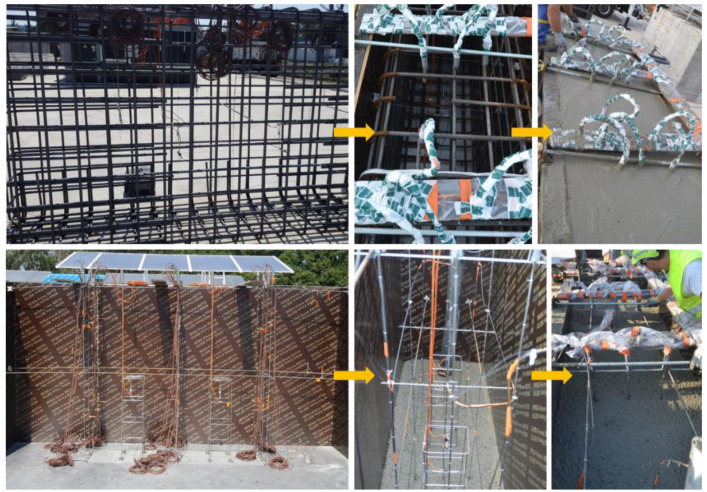
Construction steps of the two types of ODOBA blocks: RC blocks (top pictures) and NRC blocks (bottom pictures).

Figure 4 illustrates the manufacturing steps for RC blocks (top pictures) and NRC blocks (bottom pictures).

Several precautionary measures were taken to ensure a successful pouring and demolding of the NRC blocks. Indeed, the curing of the concrete at an early age was optimized using a thermal insulation device, which slowed down the thermal exchanges with the external environment. The NRC block was thus protected from extensive surface cracking that may result from excessive temperature gradient in the absence of steel reinforcement.

For the blocks intended to develop DEF, thermal conditioning was applied to the block directly after concrete pouring. This thermal treatment was carried out using a mobile steaming unit which was placed directly around the block. This steaming unit can reach a maximum temperature of 80 °C at a relative humidity (RH) of 100% and has a maximum volume capacity of approximately 50 m^3^. The steaming unit is also equipped with sensors, ventilation, and misting circuits. During the thermal treatment, the temperature within the block was continuously monitored with the embedded thermocouples (Figure 2) in order to compare the temperature of the set point imposed to the steaming unit with the values actually reached in the various regions of the block, and then possibly modulate this set point.

**Figure 5:**
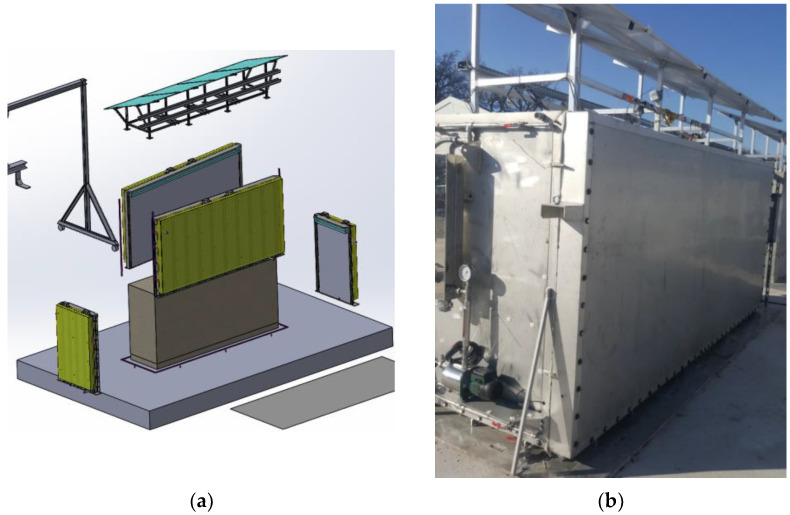
(**a**) Scheme of the pool setup used for the immersion of ODOBA blocks; (**b**) Picture of a pool in operation.

After those preliminary operations, the various blocks were subjected to different types of ageing environments in order to initiate and possibly accelerate the concrete pathologies:

- The blocks intended for natural ageing were exposed to the weather conditions on the platform.
- The blocks intended for accelerated ageing were exposed to water immersion/drying cycles. A specific device was designed to perform these ageing cycles (Figure 5). It consists of a pool filled with water, which can be heated up to 40 °C. The pool’s set points were programmed and continuously controlled by a centralized supervision system. The accelerated immersion/drying cycle was based on a two-month characteristic period: the block was immersed in water for one month, during which the temperature values recorded by the embedded thermocouples were around 25 °C, then the block was exposed directly to the outdoor environment for the next month. In order to achieve this alternation of immersion/drying, the pool can be dismantled and reassembled. Indeed, as shown in Figure 5a, the four sides of the pool are easily put in place to form a watertight structure. Once filled, the pool provides a homogeneously heated water layer around the block, thanks to an integrated water circulation system (Figure 5b). In addition, an effluent treatment station allows for monitoring the quality of the water used in the pools (pH, chemical composition).

## 3. Design of the DOFS Instrumentation

Depending on the schedule of the block instrumentation (before or after concrete pouring), the DOFS was either embedded in the concrete or bonded to the external surface of the blocks. For the embedded DOFS configuration, the instrumentation was installed before concrete pouring, and the internal trajectory of the DOFS was specific to each type of block (RC or NRC). Indeed, this trajectory was optimized in function of the supporting frames available in the block, and also for limiting risks of displacement/damage of the DOFS cable during pouring operation and for minimizing subsequent measurement disturbances along the sensing line. For the bonded DOFS configuration, the DOFS instrumentation was installed after pouring the concrete and hardening the blocks.

### 3.1. Instrumentation Scheme with Bonded DOFS

An experimental procedure was designed for bonding DOFS cables to the external surface of the existing ODOBA blocks.

**Figure 6:**
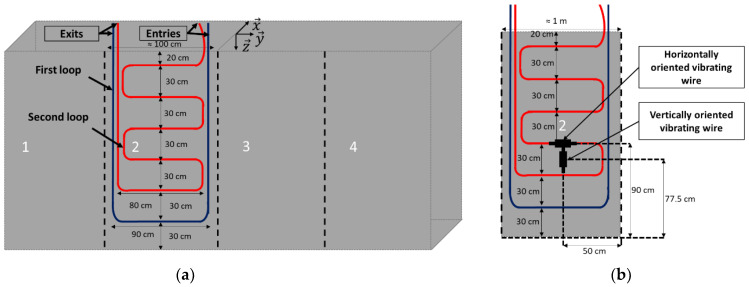
(**a**) Instrumentation scheme with bonded DOFS (**b**) Detail of the instrumented zone showing the two DOFS loops, and projection view of the embedded vibrating wires used for comparison.

One of the two larger sides of the block, with a surface area of 4 × 2 m^2^, is first divided into four identical zones (zones 1–4, see Figure 6a). Zone 2 is dedicated to receiving the bonded DOFS. The instrumentation scheme consists of two loops of DOFS cable.

The first loop (represented in blue color in Figure 6) follows the two vertical borders of zone 2 and has a horizontal portion about 30 cm above the ground level. This loop allows for the collection of the strain profiles at the two vertical borders of the instrumented zone 2, which can then be compared with the local measurements of the vertically oriented vibrating wire (VW) located halfway between the two vertical paths of the DOFS (see Figure 6b).

A second loop (red color in Figure 6) includes several horizontal paths at different heights of the block, the lowest being about 30 cm above the ground and the highest 20 cm from the top of the block. This second loop allows for the collection of the horizontal strain profiles over the whole instrumented zone and a comparison of the recorded values with the strain measurements from the horizontally oriented VW located in the same region of the block (see Figure 6b).

The concerned VWs are embedded 50 cm away from the surface of the block. Their locations in the ($\vec{y}$, $\vec{z}$) plane are shown in Figure 6b.

The entries and exits of the DOFS cable (for both loops) are located on the top side of the block to allow easy access to the connectors and the interrogation unit when the block is under accelerated ageing protocols.

### 3.2. Instrumentation of NRC Blocks with Embedded DOFS

**Figure 7:**
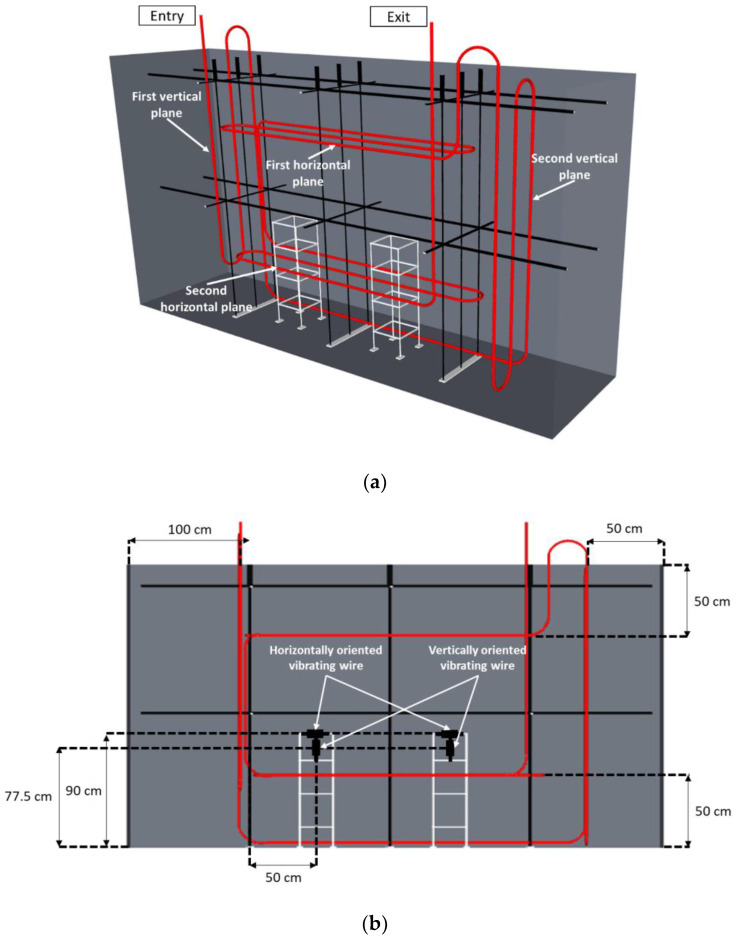
(**a**) Instrumentation scheme of NRC blocks with embedded DOFS; (**b**) Projection view showing the horizontal/vertical positions of the embedded DOFS and vibrating wire sensors.

As for the installation of thermocouples and VWs, the instrumentation of the NRC block with embedded DOFS was carried out prior to concrete pouring. In the first step, only three sides of the formwork were put in place to allow easy access to the operators. The fourth side of the formwork was only put in place after all the sensors were installed in their final locations. The internal loop of the DOFS follows a complex scheme presented in Figure 7a. As for the bonded configuration, and for the same reasons, the entry and exit of the DOFS cable are located on the upper side of the block. The DOFS path meanders first across a vertical plane perpendicular to the large faces of the block. In this plane, the DOFS cable is fixed to the metallic structure supporting the thermocouples. This first path allows for monitoring the vertical strain at several depths within the block, very close to the thin metal bars supporting the thermocouples. Such strain measurements will provide an overview of concrete swelling at various depths inside the block. In addition, the path of the DOFS cable also meanders across a second vertical plane (near the south face of the block), but here relatively far from the metallic structure bearing the thermocouples. Therefore, a comparison of the strain measurements carried out in these two vertical planes will allow for the detection of any influence of the metallic structures on the concrete swelling (possible restraining effect). It will also be interesting to compare DOFS data with local strain measurements from the two vertically oriented VWs (Figure 7b).

Two horizontal planes perpendicular to the large faces of the block are also instrumented with the embedded DOFS cable. The first horizontal plane (see Figure 7) is located at three-quarters of the block height. This horizontal DOFS path allows the strain to be measured in the upper part of the block where there is no VW. The second horizontal plane is located at a quarter of the block height, and hence the corresponding DOFS path provides horizontal strain profiles in the lower part of the block. A comparison can therefore be made between these latter DOFS data and the strain measurements from the two VW sensors oriented in the same direction and located nearby (VW are located at the height of 90 cm while the second horizontal plane is located at the height of 50 cm, see Figure 7b). Finally, just after this second horizontal path, the DOFS cable exits vertically at the top of the block.

### 3.3. Instrumentation of RC Blocks with Embedded DOFS

**Figure 8:**
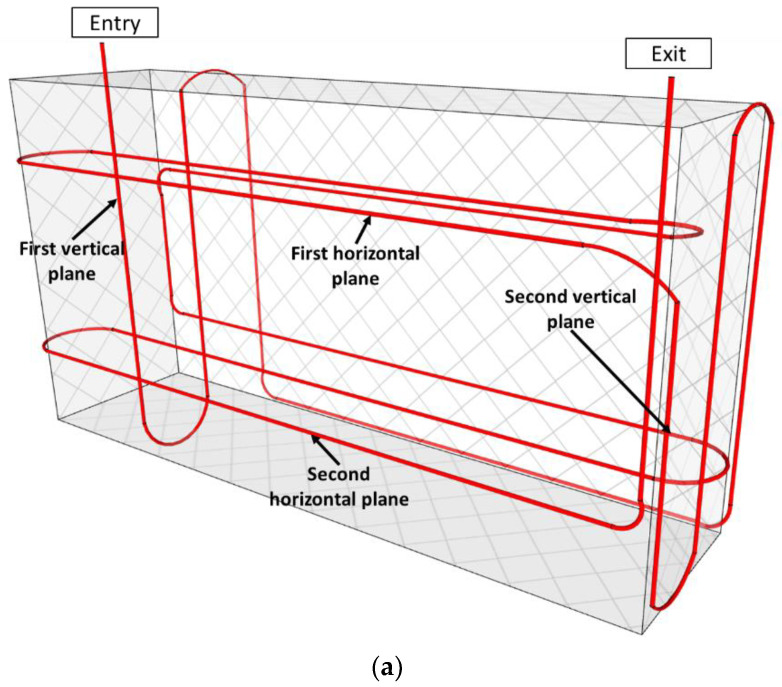

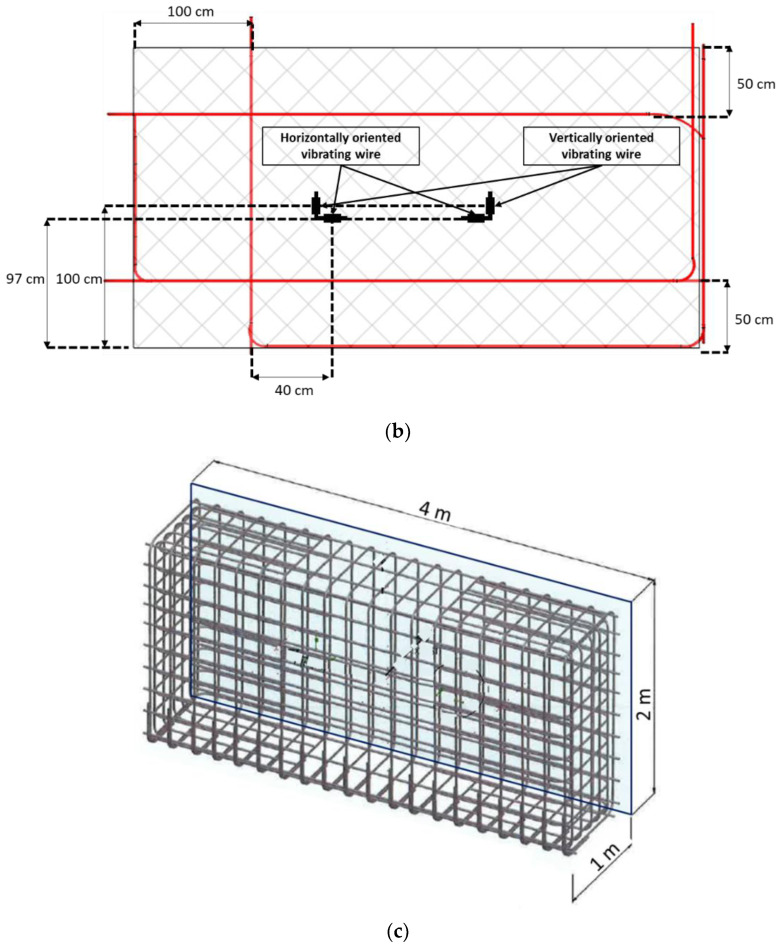
(**a**) Instrumentation scheme of RC blocks with embedded DOFS; (**b**) Projection view showing the horizontal/vertical positions of the embedded DOFS and vibrating wire sensors; (**c**) detail of the steel stirrup in RC blocks.

Unlike the previous case, the RC blocks do not contain specific metallic supports for VWs and thermocouples that could serve to fix the DOFS instrumentation. It was thus necessary to attach the DOFS cable directly to the steel stirrups and hence to adapt the internal path of the cable inside the blocks. However, the DOFS loop was still designed to pass through two vertical and two horizontal planes, as depicted in Figure 8.

Just after entering into the RC block, the DOFS cable meanders across the first vertical plane (Figure 8a). In this plane, the two descending branches of the DOFS cable are close to the steel reinforcement (see the detail of the stirrup in Figure 8c), whereas the ascending branch is distant from the steel rebars. A comparison of the strain profiles collected in these branches will thus provide relevant information about the influence of the steel reinforcement on the concrete expansion (for blocks exposed to the ageing conditions).

The DOFS then passes through the second vertical plane, where it runs upward, then downward, and upward again. In this plane, the cable path always stands close to the vertical steel reinforcement (see Figure 8c). An examination of the strain profiles in this second plane will make it possible to check symmetries of the swelling behavior in the concrete block: symmetry between the upper/lower parts of the block and symmetry with respect to the central vertical axis. Furthermore, the influence of the proximity of the block sides on concrete swelling (reflecting the influence of the external ageing environment) can also be investigated by comparing the strain measurements collected close to the sides with those obtained in the core of the RC block. Finally, a comparison of the DOFS measurements collected in the two vertical planes will also show if the swelling behavior is symmetrical on the eastern and northern sides of the block.

The DOFS instrumentation scheme of RC blocks also includes two horizontal planes located at the same heights as in NRC blocks (i.e., at ¾ and ¼ of the block height, respectively). These two instrumented levels will allow for the detection of the possible differences in terms of concrete expansion between the upper and lower parts of the exposed blocks and also compare the DOFS data with the local strain measurements from the two VWs oriented in the same direction (Figure 8a). In addition, the two horizontal plans will allow us to investigate again (i) the restraining effect of the steel reinforcement on concrete swelling, (ii) the symmetries of the block behavior, and (iii) the possible swelling gradient between the core and the sides of the block.

## 4. Installation Steps of the DOFS Instrumentation for Two Peculiar ODOBA Blocks

### 4.1. Characteristics of the DOFS Cable

**Figure 9:**
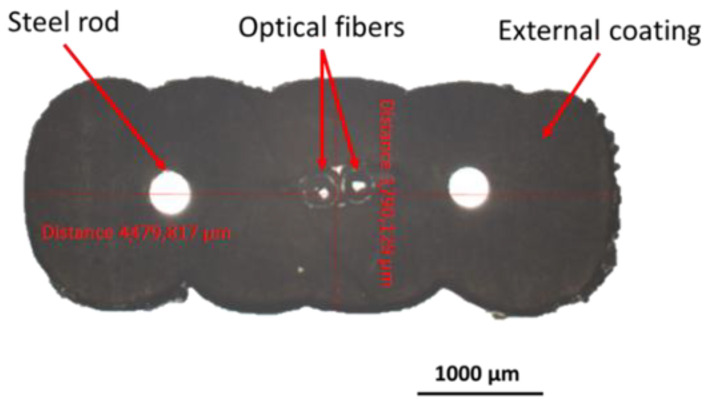
Cross-section of the DOFS cable, as observed by optical microscopy.

The DOFS cable considered in this study is the FutureNeuroTM FN-SILL-3 cable manufactured by Neubrex Co. Ltd. (Kobe, Japan) [26]. As illustrated in Figure 9, this cable is composed of two single-mode optical fibers (OFs) with an acrylate primary coating, two steel wires of diameter 0.3 mm, which ensure the mechanical reinforcement of the cable, and an external coating made of a soft elastomer.

It is worth noting that the durability of this DOFS cable was investigated in previous research under ageing conditions very similar to those considered in the present study (see [27]).

### 4.2. Instrumentation of a NRC Block Denoted “IA”

#### 4.2.1. Description of the Block IA

IA is an NRC block manufactured in May 2019 and intended for investigations under accelerated ageing conditions. The concrete mix composition contains 350 kg/m^3^ of a CEM II 42 R cement with a high sulfate and alkali content and limestone. This block was instrumented with an embedded DOFS cable (the sensors were installed in the formwork in March 2019, prior to concrete pouring). A thermal treatment was applied to this block at an early age to promote the initiation of DEF (cf. Section 2.2). Afterwards, the block was exposed to immersion/drying cycles in order to develop and accelerate this pathology.

#### 4.2.2. Instrumentation of the Block IA with Embedded DOFS

**Figure 10:**
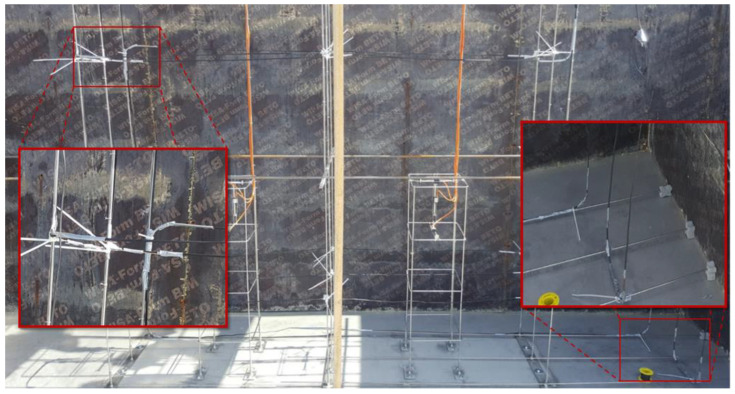
Instrumentation of block IA with embedded DOFS, and details of a metallic guide for curved parts (enclosed picture on the left side) and of the three metallic rods supporting the second vertical plane (enclosed picture on the right side).

The IA block was instrumented according to the scheme of NRC blocks presented in Section 3.2. In order to prevent displacements of the DOFS cable during concrete pouring, three horizontal metallic rods were added at the bottom of the formwork to support the second vertical plane of instrumentation (Cf. Figure 7). Metallic guides were also added to support the curved parts of the DOFS path in order to prevent the pinching of the cable and subsequent optical loss during interrogation. Pictures of the block instrumentation are presented in Figure 10.

### 4.3. Instrumentation of A RC Block Denoted “KB”

#### 4.3.1. Description of the Block KB

KB is an RC concrete block manufactured in May 2019 and also intended for investigations under accelerated ageing conditions. For this block, the concrete mix composition contained 400 kg/m^3^ of a high sulfate and low alkali CEM I 52N cement with silica-lime aggregates. Here, again, a thermal treatment was applied at an early age to initiate DEF, and the block was then subjected to immersion/drying cycles. Due to its superior sulfate content, the KB block is expected to be the most reactive among all the ODOBA blocks.

Regarding the DOFS instrumentation, the KB block was equipped both with embedded sensing cable and externally bonded cable.

#### 4.3.2. Instrumentation of the Block KB with Embedded DOFS

**Figure 11:**
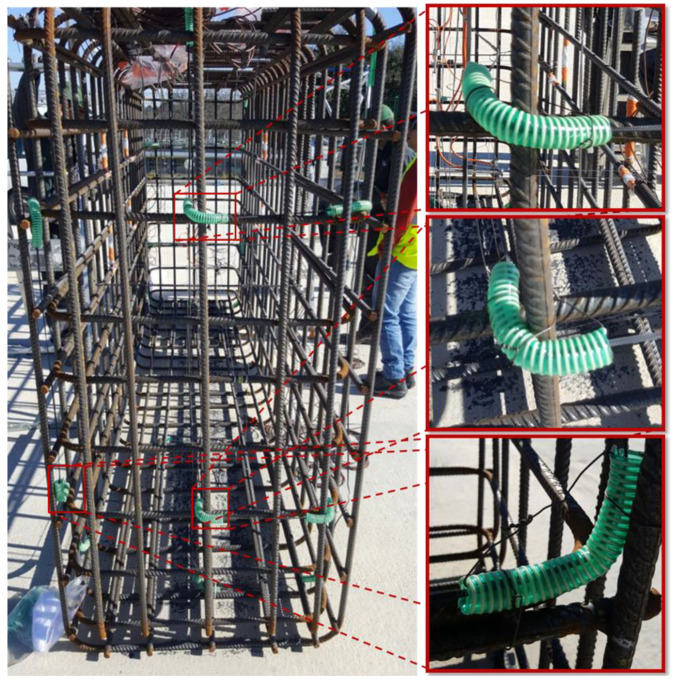
Instrumentation of block KB with embedded DOFS and details of cable bends around the stirrup (pictures on the right side).

The KB block was equipped with an embedded DOFS cable according to the instrumentation scheme of RC blocks presented in Section 3.3. In order to control the bending of the DOFS cable around the stirrup and to avoid optical losses due to pinching, guides were again used. However, for this block, the guides consisted of plastic sheaths that ensured a bending radius greater than 4 cm. Figure 11 shows some pictures taken during the instrumentation of the KB block, including details of the cable bends (on the right side).

#### 4.3.3. Instrumentation of the Block KB with Bonded DOFS

**Figure 12:**
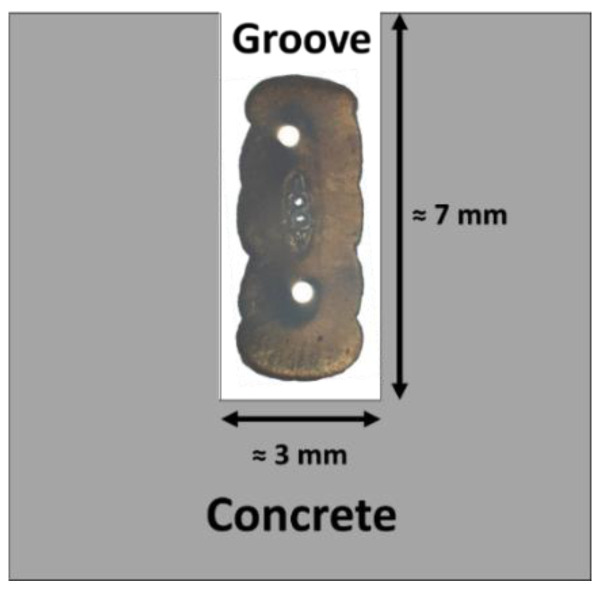
Positioning of the DOFS cable into the groove.

The block KB was also equipped with externally bonded DOFS following the instrumentation scheme presented in Section 3.1. The installation procedure consisted first in drawing the path of the cable at the surface of the block, then a groove of approximate depth 7 mm and width 3 mm was engraved along the mark using a diamond disc grinder. The groove was then cleaned, and the first layer of polymer adhesive was injected into the groove. The DOFS cable was then inserted in the groove (see the principle of insertion in Figure 12), and finally, a closing layer of adhesive was injected all along the groove. The adhesive was a bi-component epoxy system X120 commercialized by the HBM Company (Darmstadt, Germany). Its durability under the same ageing conditions as those used in this study was investigated in a previous paper [27].

The positioning of the DOFS cable into the groove, as illustrated in Figure 12, allows for the maximization of the contact surface area between the cable and concrete and hence the adhesion at the cable/concrete interface [28,29].

**Figure 13:**
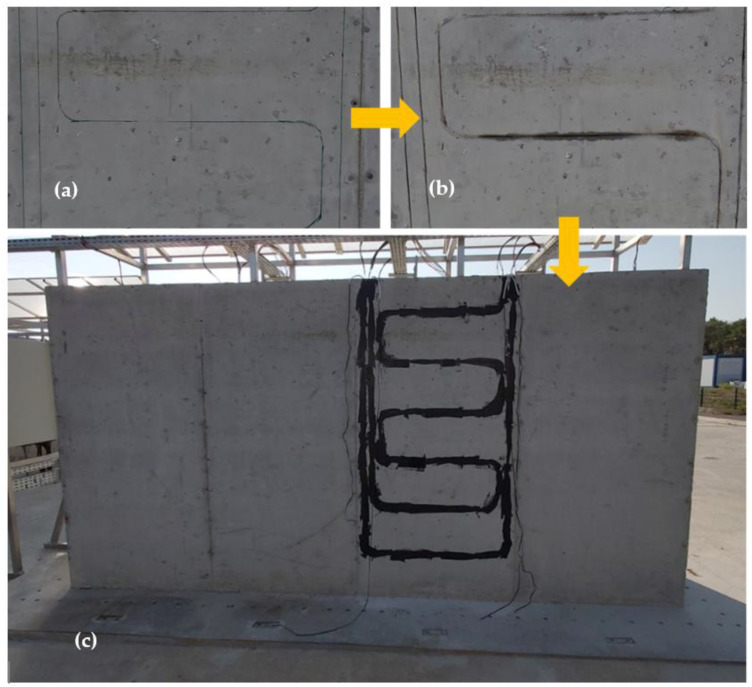
Installation steps of the DOFS cable at the surface of block KB. (**a**) Marking of the cable path at the surface of concrete (**b**) engraving of the groove along the path (**c**) insertion of the DOFS cable into the groove and application of the closing layer of X120 adhesive.

The installation steps and the final aspect of the instrumented zone are illustrated in Figure 13.

### 4.4. Schedule for the Instrumentation and the Monitoring of Blocks IA and KB

Table 1 summarizes the main actions/interventions undertaken on the two blocks: (i) the initial heat treatments and the periods of application of the immersion/drying ageing cycles, as well as (ii) the dates on which the DOFS measurements were collected using a Rayleigh interrogation unit (model OBR-4600 from Luna Innovations, Roanoke, VA, USA). A reference measurement was systematically performed to record the initial state of each block before ageing. Then, after the ageing protocol had started, interventions were planned on a regular basis (every 5 or 7 months) to collect DOFS data on the ageing blocks (the two first interventions led to measures 1 and 2, as reported in Table 1). A comparison of these data with the reference measurement is thus expected to provide information on the possible dimensional changes of the blocks during ageing, which may relate to thermal effects and/or to the development of swelling pathologies of concrete. To evaluate the contribution of swelling pathologies, it is thus necessary to remove the effects of temperature on the DOFS response. This issue is addressed in the next section.

## 5. Partial Decorrelation of the Temperature Effect from DOFS Measurements

As reported in Table 1, the strain measurements in the blocks were carried out at different times of the year. The outside temperature was thus very different on the various measurement dates, below 0 °C in winter (for measure 1) and close to 40 °C in summer (for the reference and measure 2). A decorrelation of the temperature effect should thus be performed to achieve the relevant strain values. This is even crucial for detecting the initiation of internal swelling reactions, as the expansion of concrete remains at a very low level in the early stage (for example, a threshold strain of 0.04% is usually reported in the case of DEF [30]).

Within a general framework, the spectral shift (Δ*ν_𝑅_*) measured by a Rayleigh backscatter distributed measurement interrogator depends on the strain variation (Δ*ε*) and the temperature variation (Δ*T*) as follows [22]:

Δ*ν*_𝑅_ = 𝐶_*ε*_Δ*ε* + 𝐶_𝑇_Δ*T* (1)

With:

𝐶_*ε*_: the strain sensitivity coefficient (expressed in GHz/(µm/m));

𝐶_𝑇_: the temperature sensitivity coefficient (expressed in GHz/°C).

The increment of total strain is then easily deduced from Equation (1) and from the spectral shift and temperature measurement:

Δ*ε* = (Δ*ν*_𝑅_/𝐶_*ε*_) − (𝐶_𝑇_/𝐶_*ε*_) Δ*T* (2)

In the following, the expression “direct thermal effect” refers only to the quantity (𝐶_𝑇_/𝐶_*ε*_) Δ*T* and is related to the propagation of light through the silica core (i.e., the sensing element) of the DOFS and not to the thermal expansion of the materials (concrete or silica).

Δ*ε* is composed of a part (Δ*ε*_𝑚_) resulting from the load transmitted from the host medium to the fiber core and a part (Δ*ε*_𝑇_) related (i) to the thermal expansion of the host medium transmitted to the sensor (dependent on the coefficient of thermal expansion of concrete in the present case, noted α_m_) and (ii) to the thermal expansion of the silica core of the OF (controlled by the coefficient of thermal expansion of the glass fiber, noted α_f_) which can be neglected because α_f_ << α_m_ [31].

The ratio between the two constants (𝐶_𝑇_/𝐶*ε*) is of the order of about 8.3 for standard OFs, considering the values of temperature and strain expressed in °C and in μ*ε*, respectively. In the case of a temperature variation of 10 °C between two measurements, neglecting the direct thermal effect ((𝐶_𝑇_/𝐶_*ε*_) Δ*T*) would lead to an error of 83 µm/m in the strain estimation. This direct thermal effect is, therefore, particularly noticeable for the strain measurements collected on the concrete blocks at an early age (i.e., when strain induced by concrete swelling is extremely small or close to zero). Once internal swelling pathologies have been triggered, the strain due to concrete expansion becomes much higher and can reach or even exceed 1% (10,000 µm/m). The direct thermal effect then becomes negligible at this stage.

To estimate the direct thermal effect, the thermocouple measurements were used to assess the temperature profile along the DOFS cable by selecting, along each segment of the DOFS path, the nearest thermocouple point measure. From this temperature profile and using Equation (2), the strain profile decorrelated to the direct thermal effect was finally calculated along the DOFS. Despite the relative simplicity of the proposed method, three important aspects must be specified to correctly separate the temperature effects:

- Firstly, the estimation of the temperature profile in the DOFS should rely on a relevant selection of thermocouples, depending on the type of block (NC or NRC) and the type of DOFS instrumentation (embedded or bonded DOFS cables). Indeed, the selected thermocouples must be as close as possible to the vertical or horizontal instrumentation planes in the case of embedded DOFS and as close as possible to the external surface of the block in the case of bonded DOFS;
- Secondly, it is also necessary to differentiate (i) the thermal effects leading to a variation in the strain (Δ*ε*_𝑇_) of the DOFS from (ii) the thermal effects leading to a variation in the temperature in the DOFS (not related to its thermal expansion) and thus leading to a thermal variation of the spectral shift (the term 𝐶_𝑇_Δ𝑇 in Equation (1));
- Finally, it is also necessary to validate the proposed approach and evaluate its effectiveness in removing the direct thermal effects from the raw DOFS signal. This may be achieved by comparing the strain values deduced from the proposed approach with the local strain values recorded by the conventional sensors (vibrating wires).

Concerning the second aspect, it is necessary to differentiate the global effects of temperature on the measurement of the spectral shift (𝐶_*ε*_Δ*ε*_𝑇_+𝐶_𝑇_Δ𝑇) and the effects related only to the variation of the temperature of the OF glass and thus to the way light propagates through this silica medium (𝐶_𝑇_Δ𝑇). In the present study, the objective is to measure the strain of the glass fiber (Δ*ε*), which is the sum of the two components Δ*ε*_𝑚_ and Δ*ε*_𝑇_ (i.e., the strain induced by the mechanical stresses and the strain induced by the thermal expansion/contraction of the materials). To achieve this goal through the measurement of the spectral shift, it is necessary to eliminate the influence of 𝐶_𝑇_Δ𝑇 on the measured values. The proposed method is then a partial decorrelation method of the effect of temperature, as only the effect related to the propagation of light through the silica medium is deducted from the raw data. A full decorrelation method would request to remove all the effects of temperature (including thermal expansion/contraction of materials).

Regarding the vibrating wires, it is important to note that the coefficient of thermal expansion of the steel wire (𝛼_𝑎_) is very close to that of concrete. Therefore, both materials expand/contract in the same way under a temperature change, and thermal expansion/contraction of the host medium (concrete) does not cause any additional stress to the steel wire. For this reason, the raw VW measurements (Δ*ε*_raw_) do not take into account the effects of the thermal expansion/contraction of concrete. A correction should thus be made to include this contribution and determine the global strain of the host concrete medium (Δ*ε*_total_), as expressed below [32]:

Δ*ε*_total_ = Δ*ε*_raw__𝑟_ + 𝛼_𝑎_Δ𝑇 (3)

In the present study, the calculation of Δ𝑇 is carried out by considering the temperature values recorded by the thermocouple located closest to each vibrating wire.

Taking into consideration the actual locations of the thermocouples in the blocks (see Figure 3) and the DOFS path in each instrumented block, peculiar thermocouples were selected, and their measurements were used to estimate the temperature profile along the DOFS at the different measurement dates.

For the embedded DOFS configuration, the selected thermocouples are located in the middle wire of each metallic structure (in the case of NRC blocks) or are supported by steel reinforcement (in the case of RC blocks) at different heights. These selected thermocouples allow for an estimation of the temperature of the DOFS cable in the different instrumentation planes (vertical and horizontal planes defined in Figure 7 and Figure 8).

For the bonded DOFS configuration, considering that the DOFS instrumented face was exposed to sunlight, only the thermocouples closest to this surface were selected. These thermocouples are located at a single height (mid-height of the block). The names of the thermocouples and the temperatures recorded during each intervention are listed in the various tables of Appendix A.

## 6. Analysis of the DOFS Strain Profiles on Ageing Blocks IA and KB—First Results after a Few Months of Exposure

In this section, several strain profiles collected along the DOFS loops of the blocks IA and KB are presented and analyzed. These profiles were recorded over the first months of exposure of the blocks to the immersion/drying cycles (measurement dates 1 and 2 in December 2019 and July 2020, respectively—see Section 4.4. and Table 1). It has been noted that the temperature effect has already been subtracted from these profiles using the procedure detailed in Section 5 (subtraction of term (𝐶_𝑇_/𝐶_*ε*_) Δ*T* in Equation (2)). For each segment of the embedded or bonded DOFS cable, the strain profile was compared with strain measurements from the nearest vibrating wires (Δ*ε*_total_ obtained after correction of raw data—see Equation (3)).

The spatial resolution of the Rayleigh interrogator was set at 5 cm, which is sufficient to obtain a quasi-continuous strain profile along the DOFS line. It should be noticed that DOFS were interrogated from both ends (entry and exit displayed in the schemes of Figure 6, Figure 7 and Figure 8) when possible. In addition, three different measurements were performed at the entry and exit of the block at each intervention in order to analyze and confirm the repeatability and consistency of the DOFS measurements. However, most strain profiles presented in this section are those resulting from the interrogation performed at the entry of the DOFS cable. Nevertheless, when an important optical loss was locally detected on the sensor path (due to the pinching of the sensor during the manufacturing of the block or to the stress concentration between the two measurement times, for instance), data obtained by interrogation from the exit of the DOFS cable were used to reconstruct the strain profile along the entire path length.

### 6.1. Strain Measurements along the DOFS Cable Embedded in Block IA

**Figure 14:**
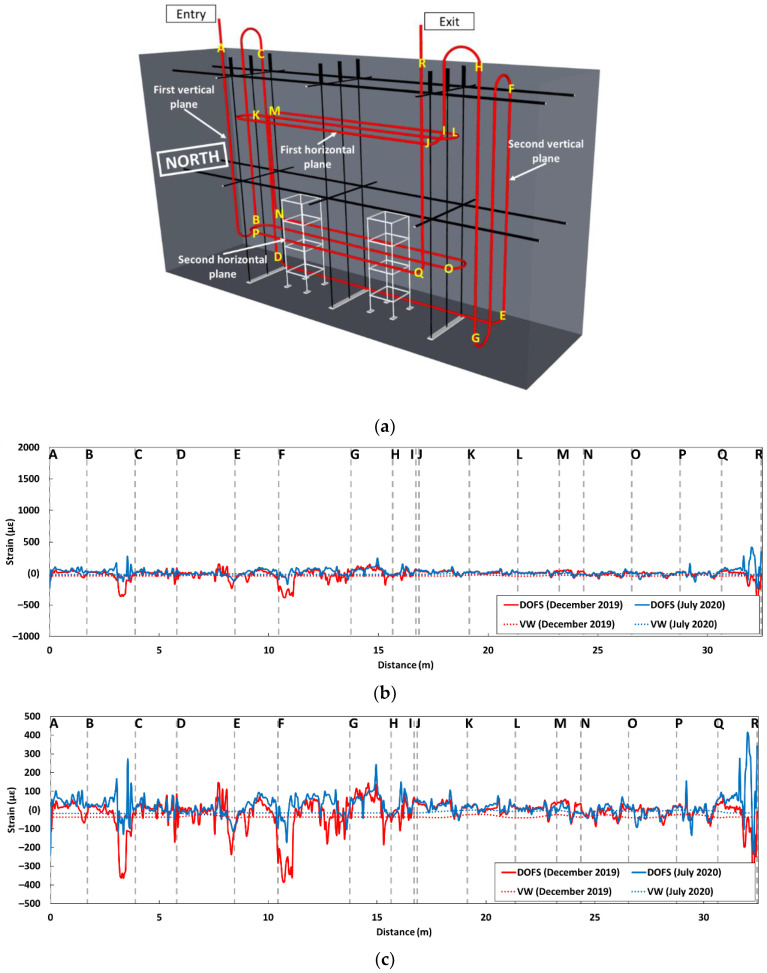
(**a**) Instrumentation scheme of the block IA with embedded DOFS, in which segments of the DOFS path are identified with alphabet letters; (**b**) Strain profiles along the DOFS (in solid lines) together with VW measurements (dotted lines); (**c**) Strain profiles at magnified y-scale.

Figure 14 presents the strain profiles recorded by the DOFS embedded in the block IA, together with strain data from the nearest VWs (Cf. location of the VWs in Figure 7b).

In Figure 14a, the instrumentation scheme of this NRC block is recalled, and the different segments of the DOFS path are identified with alphabet letters in order to facilitate the analysis of the experimental profiles.

Figure 14b displays the strain profiles using a large y-scale (*ε*-scale) that can be used in the following to compare the profiles of the two blocks IA and KB. In Figure 14c, the same profiles are displayed using a magnified scale that is more adapted to the low strain values recorded on block IA. The segments of the DOFS cable identified with letters on these profiles correspond to those previously defined in Figure 14a.

The strain profiles at both measurement times (December 2019 and July 2020) provided continuous signals along the entire length of the embedded DOFS and included the data collected from the two vertical and the two horizontal instrumentation planes (Cf. Figure 14a). The strain values recorded by the DOFS are found to be consistent with those recorded by VWs (on each considered segment of the DOFS cable, the strain values are compared to those of the nearest VW).

Although the reference measurement was performed in the middle of summer (July 2019, see Table 1) and the first measurement in December 2019, the corresponding strain profile (solid red line in Figure 14b,c) does not evidence any contraction due to the temperature drop between these two periods. This result can be explained by the fact that the first measurement (December 2019) was taken while the IA block was in the immersion pool (wet phase of the immersion/drying cycle, at a controlled temperature), and the average temperature within the block was around 25 °C (see Appendix A). This immersion thus reduced the effect of the thermal gradient between the reference measurement (temperature of the block ~32 °C, see Appendix A) and the first measurement. Moreover, the hydric swelling of the block under water immersion may also have compensated for the possible contraction due to the variation in temperature.

At the second measurement time in July 2020, the overall strain level was still very low, showing that there was no contraction/expansion of the block, consistent with the small temperature variation between the reference measurement in July 2019 (25 °C) and the measurement in July 2020 (approx. 29 °C, see Appendix A). Furthermore, this rather flat profile shows that the swelling pathology had not developed yet in block IA. This result suggests that the kinetics of swelling reactions is slow for the concrete formulation used in block IA. This is also consistent with residual expansion tests (LPC 67 test protocol [33]) carried out on core samples taken from the block, which did not reveal a sign of expansion due to the swelling pathology.

### 6.2. Strain Measurements along the DOFS Cable Embedded in Block KB

**Figure 15:**
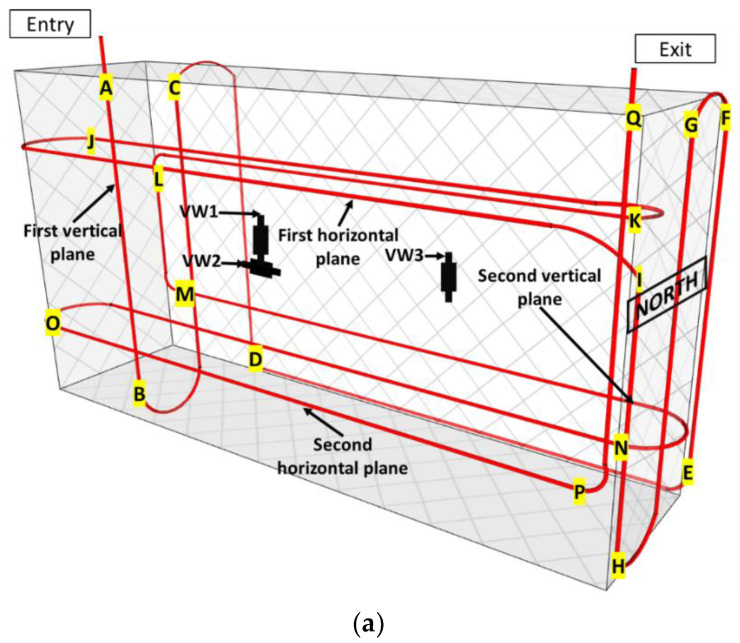

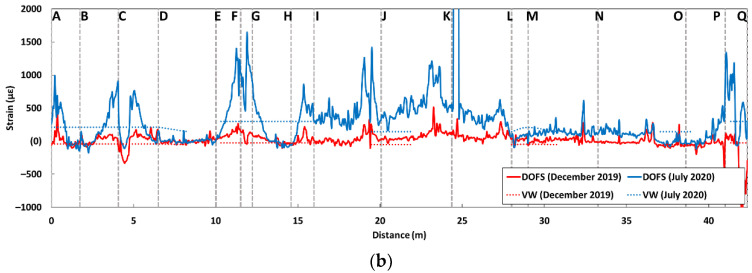
(**a**) Instrumentation scheme of the block KB with embedded DOFS, in which segments of the DOFS path are identified with alphabet letters; (**b**) Strain profiles along the DOFS (in solid lines) together with VW measurements (dotted lines).

Figure 15 displays the strain profiles recorded by the DOFS embedded in block KB, together with the strain values recorded by the three vibrating wires (VW1, VW2, and VW3, see Figure 15a) operational at the time of the measurements and located near the DOFS.

Here, again, the instrumentation scheme of this RC block is recalled in Figure 15a, and the different segments of the DOFS loop are identified using alphabet letters. Figure 15b displays the strain profiles using a large y-scale (*ε*-scale) similar to that of Figure 14b.

The results presented in Figure 15b show a satisfactory agreement between the measurements of the DOFS and the strain values recorded by the three vibrating wires. The discontinuity in the VW strain curve is related to the absence of vertical vibrating wire measures in the northern part of the block. It should be recalled that the reference DOFS measurement in this block was performed in July 2019 (while the temperature of the block was around 33 °C, see Appendix A) and that measurement 1 was performed in December 2019 (solid red line in Figure 15b) when the block was under immersion (temperature of the block around 25 °C). Therefore, the first strain measurements obtained in July do not reveal any significant contraction of the block due to the low-temperature gradient and the hydric swelling of the block concrete (same trend as previously discussed for block IA).

At the time of the second measurement (July 2020), the DOFS sensors and the vibrating wires both provided significantly larger strain values, and some peaks were recorded by the DOFS as well. These strain peaks are mainly concentrated along the I-J-K-L segments, thus at the level of the first horizontal plane located at 3/4 the block height. The strain values also increase around the A, C, F, G, P, and Q locations, i.e., at the points of curvature of the DOFS cable, rather in the vertical part and often in the upper part of the block. Overall, the average strain values are of the order of 300 μm/m (0.03%), and values around 1000–1500 μm/m (0.1–0.15%) are measured in some zones of stress concentration. These peak strains are much larger than the threshold expansion level of 0.04% commonly admitted for DEF [30] and suggest that this pathology has started to develop in the block KB at the time of the measurement in July 2020. This result was supported by the complementary residual expansion tests (method described in [33]) performed on the core specimens extracted from the KB block at the height of 1.35 m (upper part of the block), which showed expansion levels up to 7500 µm/m (0.75%), hence confirming the development of swelling pathologies. From this part, it can be concluded that the concrete formulation used in the KB block shows the higher kinetics of the swelling reaction compared to the composition of the IA block and that the embedded DOFS instrumentation is able to detect in situ the related concrete expansion.

### 6.3. Strain Measurements along the DOFS Cable Externally Bonded to the Block KB

**Figure 16:**
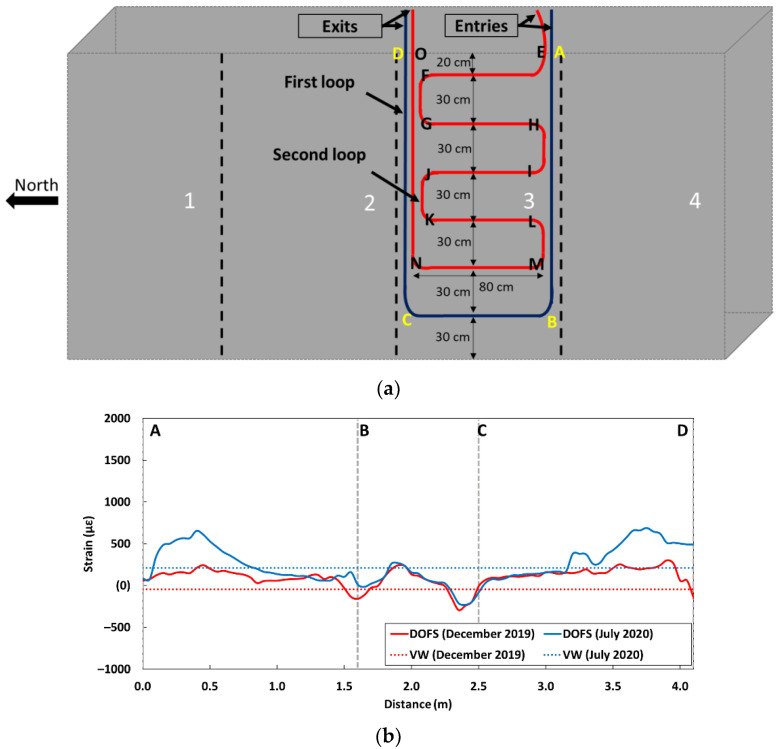

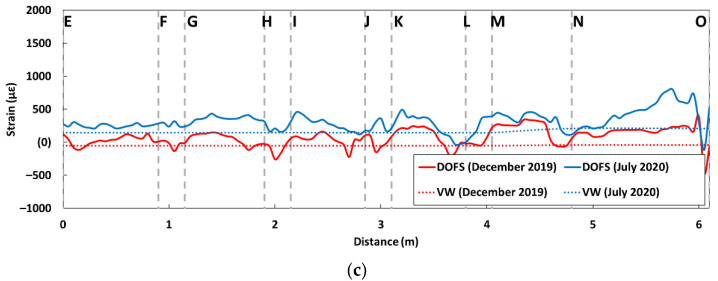
(**a**) Instrumentation scheme of the block KB with bonded DOFS, in which segments of the two DOFS loops are identified with alphabet letters; Strain profiles along the first loop (**b**) and the second loop (**c**) of the bonded DOFS cable (solid lines), together with the strain data from the nearest VWs (dotted lines).

Figure 16 presents the strain profiles recorded by the DOFS bonded to the surface of the KB block. Here, again, the instrumentation scheme is recalled in Figure 16a, together with the identification of the various segments of the DOFS cable. The strain profiles collected along the first DOFS loop and VW measurements are both displayed in Figure 16b, while the strain profiles along the second loop and corresponding VW data are shown in Figure 16c.

Overall, the results confirm the previous trends provided by the DOFS embedded in the same block:

- The results of the first measurement date in December 2019 do not show any significant contraction of the block since the block was immersed in water during this first measurement;
- A significant increase in the average strain level is observed within the block at the second measurement date in July 2020. The strain values are slightly higher in the vertical direction and in the upper part of the block (segments AB and CD of the first loop, segment NO of the second loop). This effect does not result from a thermal expansion of the block and can be attributed again to the development of the DEF pathology.

It should be noted that the strain values observed for the bonded DOFS are of lower amplitude than those obtained for the embedded DOFS. This difference may relate to the influence of the polymer adhesive used for bonding the DOFS cable, as the adhesive layer usually affects the strain transfer process between the host structure and the cable [9,23]. Moreover, the profile along the second loop (see Figure 16) shows that the intensity of the strain is globally constant over the horizontal parts of the instrumented area and for all height levels.

### 6.4. Discussion on the Heterogeneous Swelling Behaviour of the Block KB

It should be recalled that the KB block has undergone a thermal treatment directly after casting and has a higher cement content compared to the IA block. It is then very likely that swelling pathology started to develop when the block was subjected to the first immersion/drying cycle (immersion phase in December 2019), and that pathology-induced effects will become progressively more observable in the upcoming measurement campaigns.

**Figure 17:**
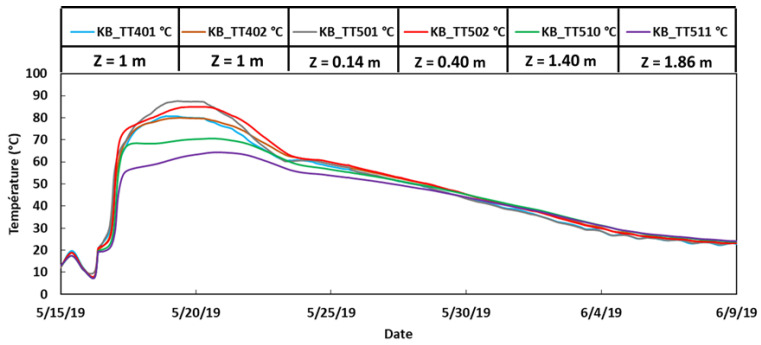
Temperatures recorded by selected thermocouples inside the block KB during the initial thermal treatment (z refers to the vertical position of the thermocouple, the origin of the $\vec{z}$ axis is located at the center of the top face of the block).

In order to analyze further the previous results provided by the DOFS instrumentation, it is interesting to examine the temperatures that were recorded within the KB block during the initial thermal treatment intended to initiate the DEF. Figure 17 shows the temperature evolutions provided by several thermocouples located at different heights of the block during the 3-week thermal treatment from 15 May to 9 June 2019. From this graph, it is found that the maximum temperature reached during the heat treatment was around 80–85 °C in the upper part of the block, whereas it was much lower (around 65–70 °C) in the bottom part of the block. Considering this vertical temperature gradient at an early age, and hence the effective thermal energy gradient created [34], it could be expected that the DEF pathology would develop first in the upper part of the KB block. This is very consistent with the strain measurements provided by the embedded DOFS in July 2020, which revealed a significantly higher expansion of concrete in the upper part of the KB block, suggesting the higher kinetics of DEF in this region of the block.

Furthermore, the temperature variations between the upper and lower parts of the block were more limited at the concrete surface during the initial thermal treatment. This can be attributed to the fact that heat diffused more homogeneously at the surface through the air layer surrounding the block. The temperature values and the effective thermal energy recorded during the heat treatment were then more homogeneous and globally higher at the surface of the block compared to the core. As the swelling pathology develops in the KB block, it is thus expected that expansion will be more homogeneous at the surface than in the depth of the block.

## 7. Conclusions

This paper presents a large-scale application of DOFS with a view to monitoring strain and detecting the swelling pathologies in massive concrete structures.

Specific protocols were first designed for the installation of the DOFS instrumentation on massive concrete blocks, considering both embedded and bonded DOFS cable configurations. The geometrical design of the sensing loop was optimized for providing a global overview of the swelling behavior in the volume and at the surface of the blocks.

These protocols were then implemented on large RC and NRC concrete blocks in the framework of the ODOBA project. These blocks, manufactured with reactive concrete formulations, were subjected to wet/dry cycles in order to accelerate the development of swelling pathologies such as DEF. The interventions were scheduled on a regular basis to collect the DOFS data using a Rayleigh interrogator, and the first results obtained over a period of one year were analyzed.

A partial decorrelation method was proposed for removing the temperature effect on the sensing zone of the DOFS (referred to as “direct thermal effect”) based on temperature measurements provided by conventional thermocouples embedded in the blocks. This method allows us to take into account the thermal gradients due to variations in the external temperature and hence to compare the DOFS data collected at different periods of the year. This decorrelation method was then validated by comparing the corrected DOFS strain profiles to the measurements provided by the nearest vibrating wires embedded in the blocks, showing a satisfying agreement.

Thanks to the embedded DOFS cable running three-dimensionally through the volume of the two blocks IA and KB, it was possible to capture the global swelling behavior of these blocks over the exposure period. Overall, the DOFS data were consistent with the local measurements from the embedded VW, but they provided continuous profiles allowing us to identify the possible differences in the behaviors between some regions or along specific directions of a given block. This is a huge advantage of the distributed DOFS technology. The main conclusions are as follows:

- Regarding the first block IA, it was found that the strain level remained very low throughout the exposure period, and no significant expansion phenomenon was detected, suggesting that the concrete formulation used for this block has low reactivity and that the kinetics of the swelling pathologies are very slow.
- Differently, for the KB block, a significant increase in the average strain level was observed in the DOFS profiles at the last intervention date, and the strain peaks were also detected in segments of the cable located in the upper part of the block. As the peak strain was higher than the characteristic threshold strain of DEF (0.04%), it could be concluded that swelling reactions had started to develop in the upper part of the block. The high reactivity of the concrete formulation used in block KB was supported by complementary residual expansion tests carried out on core specimens extracted from the upper part of the block.
- The upcoming data collection will provide more information on the complex swelling behavior of the blocks. In particular, it is expected that the effects of some parameters can be quantified, such as the presence of reinforcements, the proximity of the metallic structures carrying the thermocouples, and the curing conditions (linked to the temperature gradients).

The DOFS instrumentation bonded at the surface of the KB block confirmed the previous results from the embedded DOFS sensors, although the amplitude of the measured strains was globally lower (possibly due to the influence of the adhesive layer on the strain transfer process between the concrete structure and the fiber optic core). This surface instrumentation was able to provide a continuous strain measurement profile in two directions (vertical along the $\vec{z}$ axis and horizontal along the $\vec{y}$ axis), allowing the precise identification of the block behavior. Thanks to the high sensitivity of these sensors, it was possible (i) to measure the small thermal expansions and contractions of the blocks due to the different temperatures at the measurement times, (ii) to identify the probable effect of the hydric swelling of the blocks during the immersion cycles and (iii) to reveal an increase in the average strain level within the block KB at the last intervention date, as well as strain peaks in segments of the cable located in the upper part of this block. This latter result confirms again that swelling reactions had started to develop in this block.

As a global conclusion of this large-scale feasibility study, it can be deduced from the first results presented in the paper that DOFS have demonstrated their effectiveness for the detection and monitoring of swelling pathologies in massive concrete structures.

## Figures and Tables

**Table 1:** Schedule of the different interventions on blocks IA and KB.

Block and DOFS Configuration	Intervention Dates					
	Heat Treatment and Ageing Cycles		Interventions Related to the DOFS Instrumentation			
	May 2019	December 2019 –March 2020	March 2019	July 2019	December 2019	July 2020
IA with embedded DOFS	Heat treatment	Immersion/ drying cycles	block instrumentation	Optical connections + reference measurement	Measure 1	Measure 2
KB with embedded DOFS	Heat treatment	Immersion/ drying cycles	block instrumentation	Optical connections + reference measurement	Measure 1	Measure 2
KB with bonded DOFS			-	Block instrumentation + optical connections + reference measurement	Measure 1	Measure 2

## Appendix A

**Table A1:** Temperatures measured by selected thermocouples inside the blocks IA and KB at the different interrogation dates of the embedded DOFS instrumentation. Designation numbers of the thermocouples are the same as those presented in Figure 3. Greyed-out dates correspond to periods of immersion of the blocks in the pool.

	Selected Thermocouple														
	TT 801	TT 802	TT 803	TT 804	TT 805	TT 806	TT 501	TT 502	TT 510	TT 201	TT 202	TT 203	TT 204	TT 205	TT 206
Date of interrogation	Temperature measured by the selected thermocouple (°C)														
IA															
July 2019	33	33	32.7	32.7	32.6	32.2	33.1	33.2	32	32.7	32.7	32.5	32.4	32.3	31.7
December 2019	21.7	24.8	25.2	25.1	25.1	23.6	19.1	22.6	24.2	21.7	24.9	25.1	25.2	25.1	23.7
July 2020	30	29.7	29.2	29.1	28.8	28.2	28.7	30.3	28	29.7	29.6	29.1	29	28.7	27.7
KB															
July 2019	33.1	33.9	33.8	33.9	33.7	33	33.9	33.7	32.9	33.1	33.7	33.6	33.5	33.3	32.6
December 2019	21.3	24.4	24.8	24.7	24.8	23.8	19.3	22.4	24.2	21.5	24.4	24.8	24.8	24.6	24.0
July 2020	29.0	29.6	29.2	29.2	28.8	27.8	28.5	29.9	27.9	29.7	29.8	29.3	29.2	28.8	27.9

**Table A2:** Temperatures measured by selected thermocouples located near the surface of block KB at the different interrogation dates of the bonded DOFS instrumentation. Designation numbers of the thermocouples are the same as those presented in Figure 3. Greyed-out dates correspond to periods of immersion of the block in the pool.

	Selected Thermocouple (°C)	
	TT701	TT401
Date of interrogation	Temperature measured by the selected thermocouple (°C)	
KB		
July 2019	39.3	39.1
December 2019	25.4	25.4
July 2020	28.4	28.5
